# lncRNA CHAF1B-2 contributes to the tumorigenesis of gastric cancer by activating the Wnt/β-catenin pathway

**DOI:** 10.1038/s41598-024-84344-w

**Published:** 2025-01-02

**Authors:** Shao-Qi Tian, Jun-Jie Shen, Dao-Ping Sun, Wen-Ming Chen

**Affiliations:** 1 Department of Oncology, Jining Hospital of Xiyuan Hospital of CACMS, Jining, 272011 Shandong Province China; 2 Department of Oncology, Tianchang Traditional Chinese Medicine Hospital, Tianchang, 239300 Anhui Province China; 3 Department of Hematology, Jining No.1 People’s Hospital, Jining, 272011 Shandong Province China; 4 Department of Oncology, Jining No.1 People’s Hospital, Jining, 272011 Shandong Province China

**Keywords:** Gastric cancer, LncRNA, lnc-CHAF1B-2, Wnt/β-catenin signalling pathway, Gastrointestinal cancer, Gastric cancer

## Abstract

Lnc-CHAF1B-2, a newly discovered long noncoding RNA (lncRNA), plays a significant role in the evolution and prognosis of diverse neoplasms. However, its role in the development of gastric cancer is not yet fully understood. Using bioinformatics analysis of gastric cancer RNA-seq data from The Cancer Genome Atlas (TCGA) database, we investigated the expression of lnc-CHAF1B-2 in gastric carcinoma and its associated molecular signalling pathways. Verification through an array of in vivo and in vitro experiments—namely, EdU incorporation, flow cytometry, trans-well migration and invasion assays, subcutaneous tumour formation in nude mice, and western blot analysis—was conducted. We revealed notable upregulation of lnc-CHAF1B-2 in gastric cancer tissues. Furthermore, a positive correlation was detected between lnc-CHAF1B-2 levels and the occurrence of distant metastases in patients, which was inversely related to their prognostic outlook and survival rates. Moreover, our findings confirmed that lnc-CHAF1B-2 enhanced the proliferation, invasion, and migration of gastric cancer cells while inhibiting apoptosis both in vitro and in vivo. Mechanistically, lnc-CHAF1B-2 promoted the progression of gastric cancer through activating the Wnt/β-catenin signalling pathway. Thus, lnc-CHAF1B-2 and its regulation of the Wnt/β-catenin signalling pathway have emerged as prospective therapeutic targets in gastric cancer management.

**Supplementary Information:**

The online version contains supplementary material available at 10.1038/s41598-024-84344-w.

## Introduction

Gastric cancer, a common malignancy, originates from the epithelial lining of the stomach and comprises 95% of all malignant gastric tumours^1^. Gastric cancer is one of the leading causes of death worldwide, and its high incidence and mortality rates pose a significant threat to human health and life. According to reports, the global incidence of cancer is increasing by approximately 20 million cases annually, with gastric cancer accounting for approximately 4.9% of the total. The number of cancer-related deaths is estimated to be approximately 9.7 million cases per year, of which gastric cancer accounts for 6.8%^2,3^. The incidence and mortality rates of gastric cancer rank fifth globally among all cancer types, making it a significant threat to human health^4^. The underlying mechanisms governing the emergence and progression of gastric cancer remain incompletely understood. Research indicates that the development of gastric cancer is not attributable to a single factor but rather to an intricate process involving the interplay between genetic determinants and environmental influences associated with geographical setting, dietary practices, *Helicobacter pylori* infection, precancerous conditions, heredity, and genetic constituents^5,6^. Despite the exploration of numerous novel diagnostic and therapeutic approaches in recent years, the dismal prognosis of patients with gastric cancer persists owing to its high recurrence and metastasis rates. As a burgeoning approach in the field of oncology in recent years, targeted therapy has revolutionized the treatment of gastric cancer because of its precision in targeting specific molecular markers^6–8^. Delving into the molecular and metabolic pathways in gastric cancer cells will aid in identifying pivotal molecules closely associated with the occurrence and progression of gastric cancer, unveiling novel therapeutic targets, and comprehensively enhancing the diagnostic and therapeutic efficacy for gastric cancer.

In recent years, rapid advancements in sequencing technologies and bioinformatics have provided multifaceted means and approaches to delve into the molecular mechanisms underlying the pathogenesis and progression of gastric cancer^9,10^. The burgeoning field of long noncoding RNAs (lncRNAs) has offered novel avenues for the investigation of gastric cancer. Long noncoding RNAs (lncRNAs) are a class of noncoding RNA molecules with a length exceeding 200 nucleotides and are characterized by low expression levels and poor conservation across high expression variability^11,12^. In recent years, an increasing body of research has revealed the involvement of lncRNAs in cellular proliferation, differentiation, migration, and immune responses and their ability to drive tumour formation through interactions with other cellular components, such as DNA and proteins^11–14^. Current whole-genome sequencing has revealed a multitude of lncRNAs that are differentially expressed between normal tissues and gastric cancer. Additionally, these aberrations in their expression levels are closely correlated with the initiation, progression, invasion, metastasis, and prognosis of gastric cancer.

Lnc-CHAF1B-2 is a recently discovered lncRNA, and its presence and variation have been associated with the development and prognosis of various types of tumours. Recent investigations have revealed lnc-CHAF1B-2 to be the paramount diagnostic and prognostic biomarker for squamous cell carcinoma located in the head and neck region^15^. Additionally, lnc-CHAF1B-2 has potential as a novel prognostic biomarker for lung adenocarcinoma, promoting the growth, migration, and invasion of lung cancer cells^16,17^. Current studies suggest that lnc-CHAF1B-2 functions as an autosis and ferroptosis-associated lncRNA involved in the development of prognostic models for gastric cancer^18–20^. However, the specific role and latent mechanisms of lnc-CHAF1B-2 in the malignant progression of gastric cancer necessitate further exploration and research. This study explored the impact of lnc-CHAF1B-2 on the proliferation, migration, and invasion of gastric cancer through bioinformatics analysis and both in vivo and in vitro experiments, with a preliminary study of the molecular signalling pathways involved, to provide a new theoretical foundation and research direction for the clinical diagnosis, treatment, and prognostic evaluation of gastric cancer.

## Results

### Lnc-CHAF1B-2 is upregulated in gastric cancer and negatively correlated with the prognosis of patients with gastric cancer

**Fig. 1:**
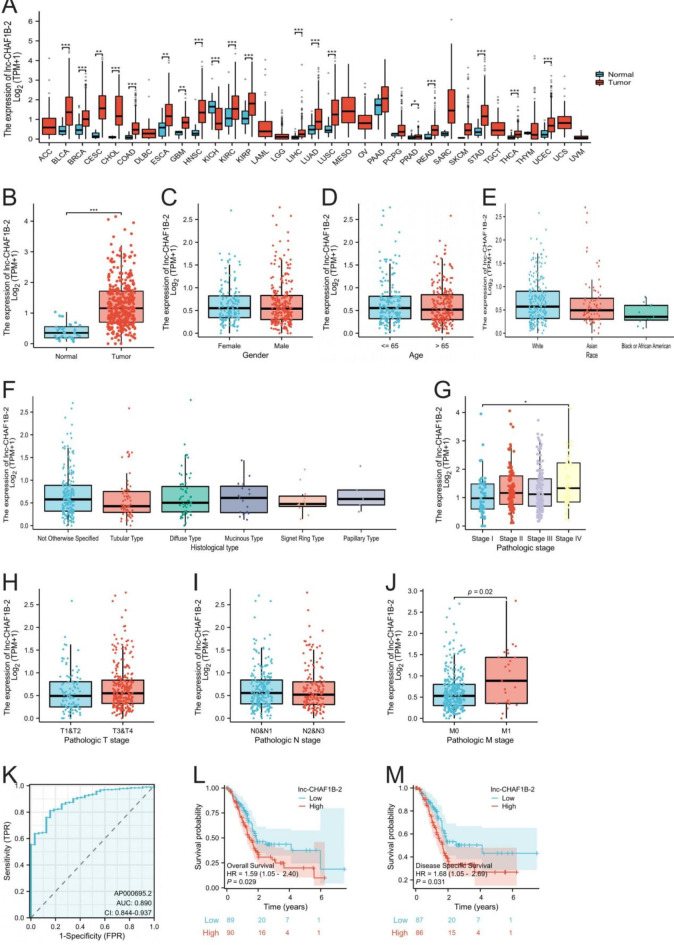
Clinical significance of lnc-CHAF1B-2. (**A**) The pivotal role of lnc-CHAF1B-2 in various cancers. (**B**) Differential expression of lnc-CHAF1B-2 between the cancer cohort and the control group. (**C**–**J**) Association of lnc-CHAF1B-2 expression with sex, age, ethnicity, pathological type, clinical stage, depth of tumour invasion, presence of lymph node metastasis, and distant metastasis in cancer patients. (**K**) lnc-CHAF1B-2 proficiently differentiated between cancerous and noncancerous tissues in gastric cancer. (**L**–**M**) Survival analysis showing that lnc-CHAF1B-2 correlated negatively with both the overall and disease-specific survival rates of patients. *P < 0.05; **P < 0.01; ***P < 0.001; ****P < 0.0001.

An analysis of data from the TCGA database revealed that the expression level of lnc-CHAF1B-2 was upregulated in 19 different types of cancer, including bladder cancer, breast cancer, cervical squamous cell carcinoma, colon cancer, oesophageal cancer, liver cancer, lung cancer, and gastric cancer, as shown in Fig. 1A. To investigate the biological effects of lnc-CHAF1B-2 in gastric cancer, we analysed its expression in gastric cancer tissues and their corresponding adjacent noncancerous tissues. The results, as illustrated in Fig. 1B, demonstrated that the expression level of lnc-CHAF1B-2 was significantly greater in gastric cancer tissues than in adjacent noncancerous tissues (*P* < 0.05).

Next, we analysed the associations between the expression of lnc-CHAF1B-2 and the clinical characteristics of gastric cancer patients. The results demonstrated that the expression of lnc-CHAF1B-2 was not significantly correlated with patients’ gender, age, ethnicity, or pathological type; however, it was associated with the clinical stage of patients. Furthermore, while there was no noteworthy relationship between lnc-CHAF1B-2 expression and the depth of tumour invasion or the presence of lymph node metastasis, a positive correlation was observed with the occurrence of distant metastasis in patients (*P* < 0.05), as illustrated in Fig. 1C–J.

We analysed the impact of lnc-CHAF1B-2 expression on the diagnosis and prognosis of gastric cancer patients. By constructing receiver operating characteristic (ROC) curves, we systematically evaluated the potential efficacy of lnc-CHAF1B-2 as a diagnostic biomarker for gastric cancer. The analysis results, as shown in Fig. 1K, revealed an area under the ROC curve of 0.890 (95% CI: 0.844–0.937), with a sensitivity of 0.81, specificity of 0.84, and accuracy of 81.3%. Furthermore, its positive predictive value was 98.4%, and its negative predictive value was 27.6%. Through Kaplan‒Meier survival analysis, we investigated the associations between the expression levels of lnc-CHAF1B-2 and the overall survival (OS) and disease-specific survival (DSS) of gastric cancer patients. The analysis results, as depicted in Fig. 1L,M, revealed a significant negative correlation between high expression of lnc-CHAF1B-2 and the OS and DSS of patients with gastric cancer (*P* < 0.05). Based on the aforementioned findings, we can draw the following conclusions: the expression level of lnc-CHAF1B-2 was upregulated in gastric cancer tissues, and it provided high efficacy as a diagnostic biomarker. Furthermore, high expression of lnc-CHAF1B-2 was significantly negatively correlated with both the overall survival and disease-specific survival of patients. These results collectively suggest that lnc-CHAF1B-2 may play a pivotal role in the pathogenesis of gastric cancer, potentially promoting disease progression.

### lnc-CHAF1B-2 enhances the malignant proliferation, migration, and invasion of gastric cancer

**Fig. 2:**
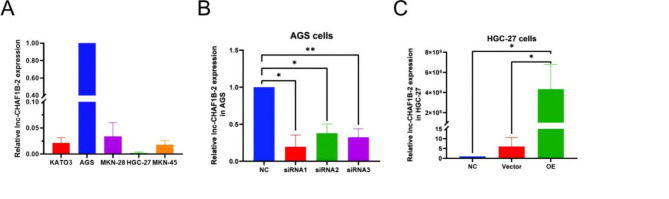
The molecular function of lnc-CHAF1B-2 was explored in HGC-27 and AGS cells. (**A**) Examination of lnc-CHAF1B-2 expression in five human gastric cancer cell lines via RT‒qPCR. The abundance of lnc-CHAF1B-2 was relatively high in AGS cells and relatively low in HGC-27 cells. (**B**) lnc-CHAF1B-2 inhibitory gastric cancer cell lines were established in AGS cells using siRNA1. (**C**) The lnc-CHAF1B-2-overexpressing gastric cancer cell line was established in HGC-27 cells using lentiviral transduction. NC: blank control group, siRNA1:small interfering RNA1, siRNA2:small interfering RNA2, siRNA3:small interfering RNA3, Vector: empty vector, OE: lnc-CHAF1B-2 overexpression.*P < 0.05; **P < 0.01; ***P < 0.001; ****P < 0.0001.

We detected the expression levels of lnc-CHAF1B-2 in five human gastric cancer cells by qPCR, and the results, as shown in Fig. 2A, revealed that lnc-CHAF1B-2 was relatively more abundant in AGS cells than in HGC-27 cells. We established three AGS inhibitory cell lines using the RFect small RNA transfection reagent. qPCR revealed a significant decrease in the expression level of lnc-CHAF1B-2 in the first inhibitory cell line (*P* < 0.05), as illustrated in Fig. 2B. The first inhibitory cell line was selected for subsequent experiments. We constructed lnc-CHAF1B-2-overexpressing HGC-27 cells via lentiviral transduction. qPCR revealed a significant increase in the expression level of lnc-CHAF1B-2 in the overexpression group (*P* < 0.05), as illustrated in Fig. 2C. Stable lnc-CHAF1B-2-knockdown and -overexpressing gastric cancer cell lines will be utilized for the assessment of biological functions, including proliferation, apoptosis, invasion, migration, and tumour formation.

### lnc-CHAF1B-2 enhances the malignant proliferation of gastric cancer cells

**Fig. 3:**
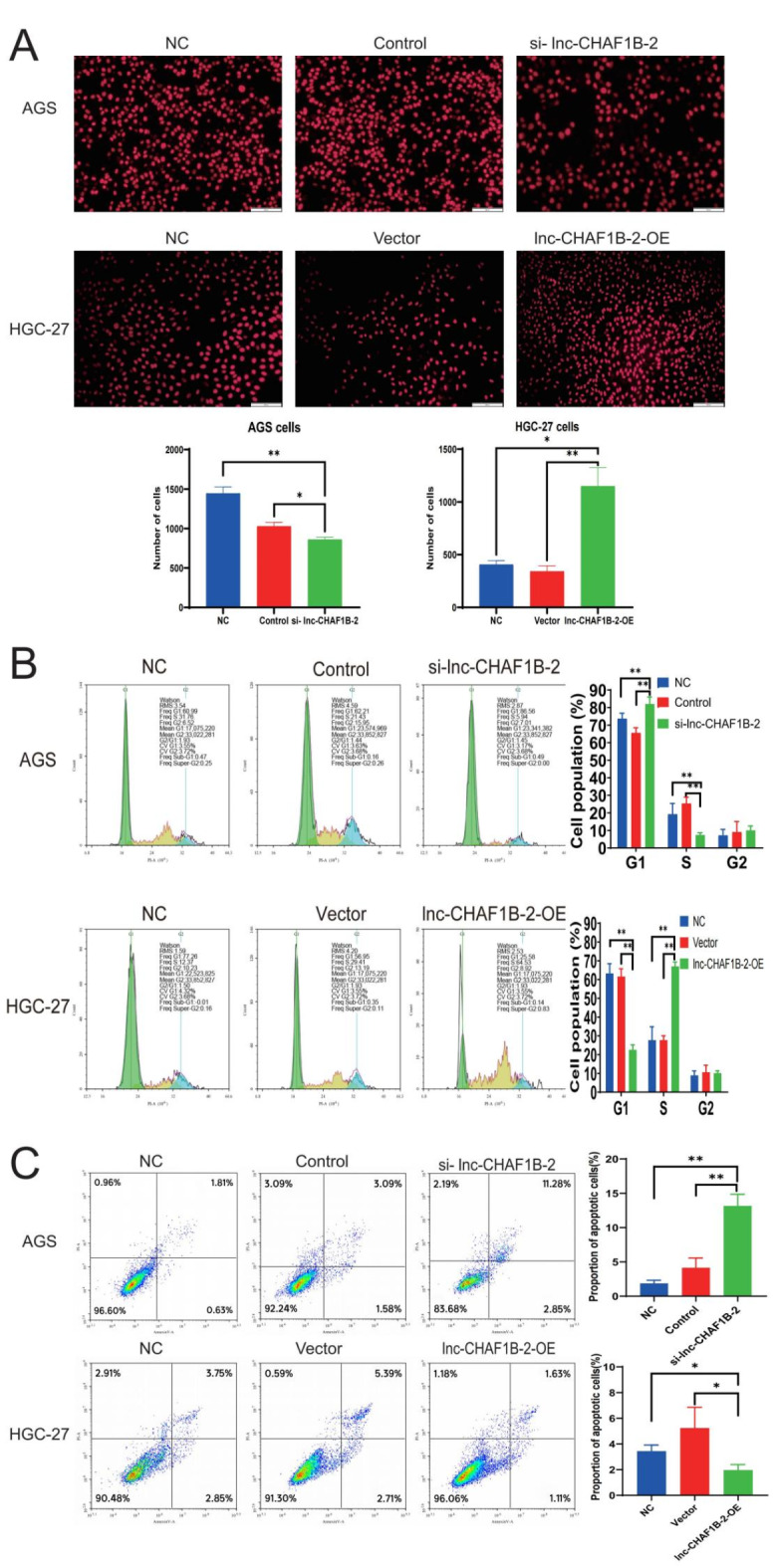
Lnc-CHAF1B-2 enhanced the malignant proliferation of gastric cancer cells. (**A**) The results of the EdU assay revealed that knockdown of lnc-CHAF1B-2 reduced the malignant proliferation of gastric cancer cells, whereas overexpression of lnc-CHAF1B-2 enhanced their malignant proliferation (scale bar, 500 μm). (**B**) Flow cytometry images of the cell cycle in gastric cancer cells in which lnc-CHAF1B-2 was knocked down or overexpressed. (**C**) Flow cytometry images of apoptosis in gastric cancer cells in which lnc-CHAF1B-2 was knocked down or overexpressed. NC: blank control group, Control: negative control, si-lnc-CHAF1B-2:small interfering RNA1, Vector: empty vector, lnc-CHAF1B-2-OE: lnc-CHAF1B-2 overexpression.*P < 0.05; **P < 0.01; ***P < 0.001; ****P < 0.0001.

First, we assessed the impact of lnc-CHAF1B-2 on the proliferation of gastric cancer cells using an EdU assay. The results revealed that inhibition of lnc-CHAF1B-2 could diminish the proliferative capacity of AGS cells, whereas overexpression of lnc-CHAF1B-2 could enhance the proliferation of HGC-27 cells, with statistically significant differences (*P* < 0.05), as illustrated in Fig. 3A.

We subsequently evaluated the impact of lnc-CHAF1B-2 inhibition and overexpression on the cell cycle progression of gastric cancer cells via flow cytometry. The results revealed that inhibition of lnc-CHAF1B-2 led to an increase in the population of AGS cells in the synthesis phase, indicating cell cycle arrest, whereas overexpression of lnc-CHAF1B-2 resulted in a decrease in the population of HGC-27 cells in the synthesis phase, suggesting accelerated cell cycle progression, as illustrated in Fig. 3B.

Thereafter, we employed flow cytometry to assess the impact of lnc-CHAF1B-2 on the apoptosis of gastric cancer cells. The results revealed that inhibition of lnc-CHAF1B-2 increased the proportion of cells in the Q2 quadrant, indicating increased apoptosis of gastric cancer cells, whereas overexpression of lnc-CHAF1B-2 slightly decreased the number of cells in the Q2 quadrant, thus suppressing cell apoptosis, as depicted in Fig. 3C.

### lnc-CHAF1B-2 enhances the migration and invasion of gastric cancer cells

**Fig. 4:**
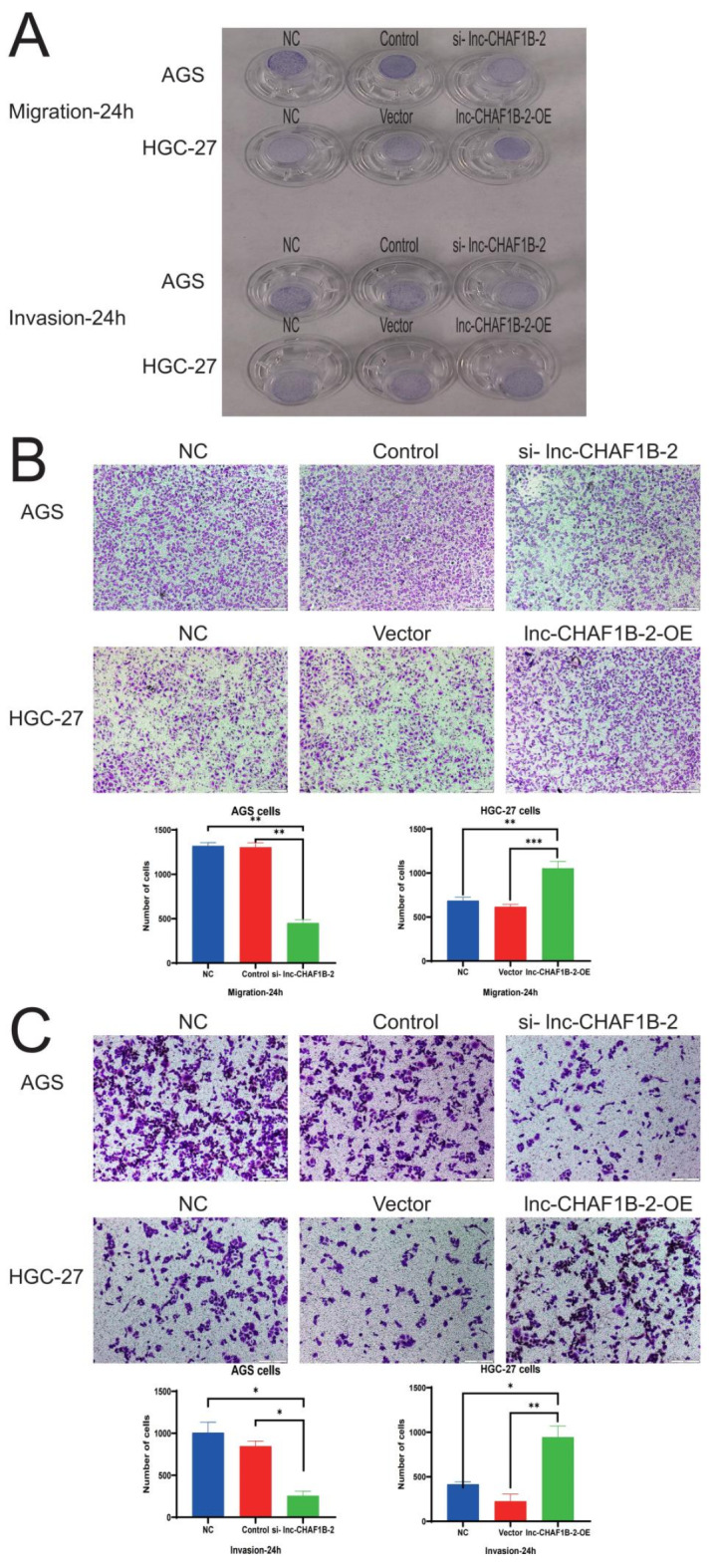
Lnc-CHAF1B-2 enhanced the migration and invasion of gastric cancer cells. (**A**) Overall picture of the trans-well migration and invasion. (**B**) Trans-well migration results showing that knockdown of lnc-CHAF1B-2 reduced the migration of gastric cancer cells, whereas overexpression of lnc-CHAF1B-2 enhanced their migration (scale bar, 200 μm). (**C**) Trans-well invasion results indicated that the reduction in lnc-CHAF1B-2 diminished the invasion of gastric cancer cells, whereas overexpression of lnc-CHAF1B-2 promoted their invasion (scale bar, 200 μm). NC: blank control group, Control: negative control, si-lnc-CHAF1B-2:small interfering RNA1, Vector: empty vector, lnc-CHAF1B-2-OE: lnc-CHAF1B-2 overexpression.*P < 0.05; **P < 0.01; ***P < 0.001; ****P < 0.0001.

We conducted trans-well migration and invasion assays to investigate the influence of lnc-CHAF1B-2 on the migratory and invasive capabilities of gastric cancer cells. The results demonstrated that inhibition of lnc-CHAF1B-2 suppressed the migration and invasion of gastric cancer cells, while overexpression of lnc-CHAF1B-2 significantly increased their migratory and invasive abilities (*P* < 0.05), as depicted in Fig. 4.

### lnc-CHAF1B-2 enhances the tumour growth in vivo

**Fig. 5:**
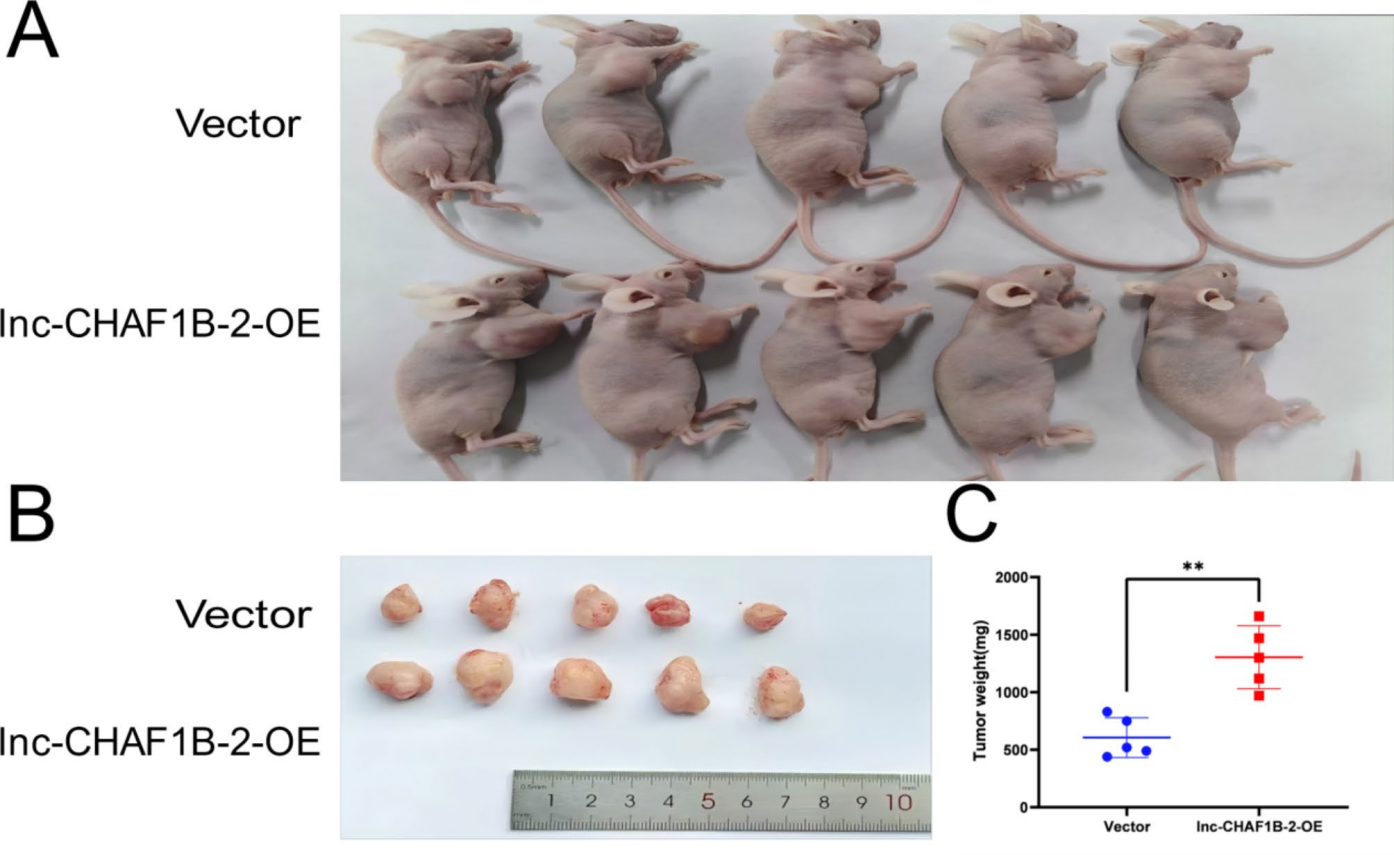
Lnc-CHAF1B-2 enhanced the tumorigenesis of gastric cancer cells. (**A**, **B**) The results of the subcutaneous tumour formation assay revealed that overexpression of lnc-CHAF1B-2 enhanced tumour formation. (**C**) Tumour weights of the mice in the different groups. Vector: empty vector, lnc-CHAF1B-2-OE: lnc-CHAF1B-2 overexpression. *P < 0.05; **P < 0.01; ***P < 0.001; ****P < 0.0001.

Finally, we conducted a subcutaneous tumour formation assay in nude mice to investigate the impact of lnc-CHAF1B-2 overexpression on the tumorigenesis of AGS cells. The results demonstrated that, compared with the control group, overexpression of lnc-CHAF1B-2 significantly promoted tumour formation in AGS cells (*P* < 0.05), as shown in Fig. 5.

### Lnc-CHAF1B-2 activates the Wnt/β-catenin signalling pathway in gastric cancer

**Fig. 6:**
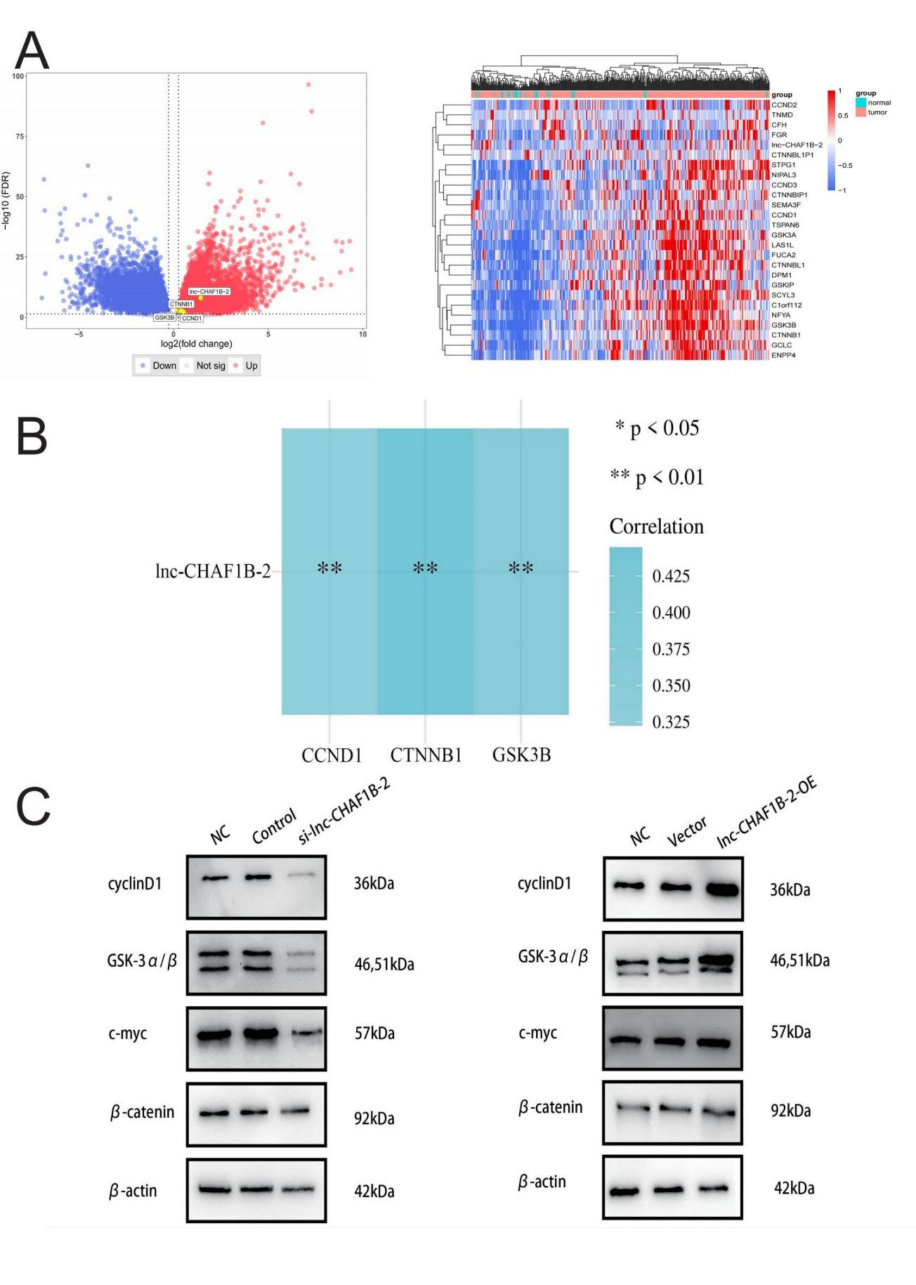
Lnc-CHAF1B-2 activated the Wnt/β-catenin signalling pathway in gastric cancer. (**A**) Volcano plot and heatmap of the DEGs which were correlated with lnc-CHAF1B-2 expression in gastric cancer. (**B**) Association of lnc-CHAF1B-2 with genes encoding pivotal proteins within the Wnt/β-catenin signalling pathway, including β-catenin, GSK-3β, and cyclin D1, which correspond to the genes CTNNB1, GSK3B, and CCND1. (**C**) Western blot results showing the protein levels of β-catenin, GSK-3α/β, cyclin D1, and c-myc in gastric cancer cells following knocking down and overexpression of lnc-CHAF1B-2. NC: blank control group, Control: negative control, si-lnc-CHAF1B-2:small interfering RNA1, Vector: empty vector, lnc-CHAF1B-2-OE: lnc-CHAF1B-2 overexpression.

Lnc-CHAF1B-2 is a recently discovered long noncoding RNA (lncRNA) without a designated gene symbol, which precludes the identification of its signalling pathway through single-sample gene enrichment analysis. In view of the extensive research conducted by our research group on the Wnt/β-catenin signalling pathway, we explored the correlation between lnc-CHAF1B-2 and genes encoding key proteins of the Wnt/β-catenin pathway, including β-catenin, GSK-3β, and cyclin D1 (corresponding to the genes CTNNB1, GSK3B, and CCND1). As shown in Fig. 6A,B, lnc-CHAF1B-2 was positively correlated with CTNNB1, GSK3B, and CCND1. Based on these findings, we tentatively hypothesized that lnc-CHAF1B-2 might exert its function in gastric cancer through the regulation of the Wnt/β-catenin signalling pathway. Through WB analysis, we detected changes in the expression of key proteins in the Wnt/β-catenin signalling pathway in gastric cancer cells following the knockdown and overexpression of lnc-CHAF1B-2. The results indicated that downregulation of lnc-CHAF1B-2 resulted in decreased protein levels of β-catenin, GSK-3α/β, cyclin D1, and c-myc, whereas upregulation of lnc-CHAF1B-2 led to increased expression of these proteins, as shown in Fig. 6C. This suggested that lnc-CHAF1B-2 influenced the initiation and progression of gastric cancer by activating the Wnt/β-catenin signalling pathway.

## Discussion

Increasing evidence suggests that epigenetic alterations, including those in lncRNAs, play a crucial role in the development of gastric cancer^21–24^. Leveraging the TCGA database, we obtained clinical information and transcriptomic data from 375 gastric cancer samples and 35 adjacent noncancerous samples. Bioinformatics analysis revealed that lnc-CHAF1B-2 expression was elevated in gastric cancer tissues and was positively correlated with distant metastases in gastric cancer patients. We subsequently examined the correlation between lnc-CHAF1B-2 expression and the survival and prognosis of gastric cancer patients and discovered a significant negative correlation between lnc-CHAF1B-2 expression levels and patient survival and prognosis. These findings indicated that lnc-CHAF1B-2 could be a prospective biomarker for predicting the prognosis of gastric cancer patients. Based on the aforementioned bioinformatics analysis results, we hypothesized that lnc-CHAF1B-2 was involved in the pathogenesis and progression of gastric cancer. To further verify the impact of lnc-CHAF1B-2 on the biological behaviour of gastric cancer cells, we used quantitative real-time PCR to detect lnc-CHAF1B-2 expression in five different human gastric cancer cell lines and discovered its presence across all the lines. We utilized siRNA inhibition technology to downregulate lnc-CHAF1B-2 expression in AGS gastric cancer cells, which presented the highest expression levels, and lentiviral transfection to upregulate lnc-CHAF1B-2 expression in HGC-27 gastric cancer cells, which had the lowest expression levels. Through a series of in vitro and in vivo experiments, including EdU proliferation assays, flow cytometry, trans-well migration and invasion assays, and subcutaneous tumour formation in nude mice, we explored the multifaceted effects of lnc-CHAF1B-2 on the biological functions of gastric cancer cells. The results demonstrated that suppression of lnc-CHAF1B-2 inhibited the proliferation, cell cycle progression, migration, and invasion of gastric cancer cells while promoting apoptosis. Conversely, overexpression of lnc-CHAF1B-2 increased the proliferation, cell cycle progression, migration, and invasion of gastric cancer cells and suppressed their apoptosis. In summary, lnc-CHAF1B-2 played a pivotal role in the progression of gastric cancer and was closely associated with its diagnosis and prognosis. Thus, further exploration of the specific regulatory mechanisms of lnc-CHAF1B-2 in gastric cancer cells is imperative to provide valuable insights for gastric cancer treatment.

The genesis of cancer originates from perturbations in the delicate equilibrium between cellular proliferation, differentiation, and programmed cell death. Within this framework, proteins integral to signalling cascades governing cell growth and differentiation are more susceptible to oncogenic transformation than other genetic elements^25^. The Wnt/β-catenin pathway represents a pivotal nexus in cellular signalling, exerting crucial regulatory control over fundamental cellular functions such as proliferation, differentiation, and apoptosis. Anomalies in its activation or inhibition are intricately linked to the process of carcinogenesis^26–29^. By exploring the correlation between lnc-CHAF1B-2 and key proteins in Wnt/β-catenin D1, which correspond to the genes CTNN GSK3B and CCND1, we hypothesized that lnc-CHAF1B-2 might promote the development and progression of gastric cancer through activation of the Wnt/β-catenin pathway. Experimental validation through Western blot analysis revealed that downregulating lnc-CHAF1B-2 expression attenuated the levels of key proteins in the Wnt/β-catenin pathway, including β-catenin, GSK-3β, cyclin D1, and c-myc, whereas overexpression of lnc-CHAF1B-2 upregulated these proteins. In conclusion, lnc-CHAF1B-2 orchestrated gastric cancer development by modulating the activation of the Wnt/β-catenin signalling pathway.

LncRNAs participate in the occurrence and progression of tumours by regulating signalling pathways related to tumour cell proliferation, apoptosis, migration, invasion, epithelial‒mesenchymal transition (EMT), autophagy, and other processes^13,21–24^. Therefore, targeting lncRNAs holds promising potential as a therapeutic strategy for gastric cancer. In our study, we observed significant upregulation of lnc-CHAF1B-2 expression in gastric cancer tissues. We found that lnc-CHAF1B-2 activated the Wnt/β-catenin signalling pathway, promoting biological processes such as proliferation, cell cycle progression, invasion, and metastasis in gastric cancer cells. These findings highlight the potential of lnc-CHAF1B-2 as a predictive biomarker for gastric cancer development and as a novel therapeutic target. This discovery establishes an experimental foundation for future in-depth research in this area.

## Methods

### Data extraction

The bioinformatics analysis data presented herein were derived from the TCGA (The Cancer Genome Atlas) database (https://portal.gdc.cancer.gov/). Data were downloaded from the TCGA database, resulting in clinical and transcriptomic information for 375 gastric cancer samples and 35 adjacent normal samples. The analysis was conducted using R software (version 4.1.0), incorporating a suite of R packages, including “ggplot2”, “pROC”, “heatmap”, “survminer”, “survival”, “clusterProfiler” and “org.Hs.eg.db”.

### Cell culture

The gastric cancer cell lines HGC-27 and MKN-28 were procured from Beijing Beina Chuanglian Biotechnology Research Institute, while the gastric cancer cell lines AGS, KATO-3, and MKN-45 were obtained from Wuhan Pricella Biotechnology Co., Ltd. The HGC-27 cell line was cultured in RPMI-1640 medium supplemented with 20% foetal bovine serum; the AGS cell line was cultured in F12 medium supplemented with 10% foetal bovine serum; and MKN-28, KATO-3, and MKN-45 were cultured in RPMI-1640 medium supplemented with 10% foetal bovine serum. The cells were incubated at 37 °C in a 5% CO_2_ incubator and regularly tested to confirm their mycoplasma-free status.

### Establishment of inhibitory cell lines in gastric cancer cells

First, gastric cancer cells were seeded at 70% density in 6-well plates. Next, the transfection reagent was diluted by mixing 240 µl of Opti-MEMI medium with 10 µl of RFect transfection reagent, and the interference sequence diluent was prepared by combining 245 µl of Opti-MEMI medium with 5 µl of siRNA. Both solutions were then combined and incubated at room temperature for 20 min. Finally, 1.5 ml of complete culture medium was added, and the mixture was incubated in a 37 °C incubator with 5% CO_2_ for 72 h before proceeding to subsequent experiments.

### Establishment of overexpressing gastric cancer cell lines

First, gastric cancer cells were seeded at 20% density in 6-well plates. Next, the infection solution was prepared by mixing 40 µl of infection enhancer P solution, 960 µl of complete culture medium, and 2.2 µl of lentivirus at a specified ratio. One millilitre of infection mixture was added to each well, and the mixture was incubated for 72 h; subsequently, the lentiviral infection efficiency was assessed using quantitative PCR. To select the HGC-27-overexpressing cell line, the cells were cultured in complete medium containing 10 µg/mL puromycin for 48 h, and complete medium supplemented with 8 µg/mL puromycin was used for selection of the AGS-overexpressing cell line. The culture was continued with complete medium at half the selected puromycin concentration for subsequent experiments.

### Quantitative PCR (qPCR)

The reaction primers were designed by Wuhan Pricella Biotechnology Co., Ltd. Total RNA was extracted from gastric cancer cells using the Quick RNA Extraction Kit supplied by Fastagen Biotech, Shanghai. Following reverse transcription of the total RNA, reaction mixtures were prepared on ice and subjected to quantitative real-time PCR to analyse the Ct values of the samples. Additionally, the specificity of the resulting PCR products was validated using melting curve analyses.

### EdU assay

The proliferation of AGS and HGC-27 gastric cancer cells was assessed using an EdU assay kit (Beyotime Biotechnology, Shanghai). First, gastric cancer cells were seeded at a density of 1 × 10^5^ cells/well in 6-well plates for 24 h. Subsequently, 10 µM EdU reagent was added to the culture medium, and the cells were incubated at 37 °C with 5% CO_2_ for 2 h. After incubation, the cells were fixed, washed, and stained with Click reaction mixture. Finally, the number of EdU-positive cells was observed under a fluorescence microscope.

### Trans-well migration assays

First, gastric cancer cells were seeded at a density of 1 × 10^4^ cells/well in trans-well chambers and incubated in 24-well plates at 37 °C with 5% CO_2_ for 24 h. Next, the chambers were fixed in methanol at room temperature for 30 min and stained with crystal violet solution for 30 min. Finally, the cells were observed and photographed under a 10× microscope.

### Trans-well invasion assays

First, 100 µl of Matrigel (Corning Incorporated, America) was added to the trans-well chambers, which were then incubated for 4 h. The steps of the trans-well migration assays were then repeated.

### Flow cytometric analysis of the cell cycle

First, 1 × 10^5^ gastric cancer cells were transferred to 1 ml of 70% ethanol and fixed at 4 °C for 12 h. Then, the Cell Cycle and Apoptosis Detection Kit (from Beyotime Biotechnology, Shanghai) was used to prepare the propidium iodide staining solution, and staining was performed in the dark for 30 min. Finally, the cell cycle analysis was performed using a flow cytometer.

### Flow cytometric analysis of apoptosis

A total of 1 × 10^5^ gastric cancer cells were collected, and an Annexin V-FITC Apoptosis Detection Kit (from Beyotime Biotechnology, Shanghai) was used to prepare the Annexin V-FITC mixture. This mixture was then applied to the gastric cancer cells under dim light for a 20-minute staining period. Flow cytometry was subsequently used to assess the apoptotic status of the cells.

### Western blot analysis

Initially, a cellular protein extraction kit (from Wuhan Pricella Biotechnology Co., Ltd.) was used to extract total proteins from both gastric cancer tissues and cells. Following separation via SDS‒polyacrylamide gel electrophoresis (SDS‒PAGE), the total protein from each sample was transferred onto polyvinylidene fluoride (PVDF) membranes. At room temperature, the membranes were blocked with protein-free rapid blocking solution (from Beijing Solarbio Science & Technology Co., Ltd.) for 15 min and then incubated overnight at 4 °C with primary antibodies targeting the following specific proteins: β-actin (1:1,000; Affinity, USA), β-catenin (1:1,000; CST, USA), GSK-3α/β (1:1,000; CST, USA), cyclin D1 (1:1,000; CST, USA), and c-myc (1:1,000; CST, USA). After a 2-hour incubation with secondary antibodies, the protein bands were subsequently visualized using a chemiluminescence (ECL) kit (from Beyotime Biotechnology, Shanghai).

### Subcutaneous tumour formation assay in nude mice

Ten 4-week-old male BALB/c-nu mice were housed under specific pathogen-free (SPF) conditions at Chenxin Pharmaceutical Experimental Animal Center. Ten nude mice were randomly divided into two groups, with each group receiving a subcutaneous injection in the axilla of 0.1 ml of cell suspension with a concentration of 2 × 10^7^/ml AGS or AGS-overexpressing cells. Thirty days later, the nude mice were euthanized by cervical dislocation, and the tumours were surgically excised, photographed, weighed, fixed, and subjected to immunohistochemistry and Western blot analysis for the detection of pathway protein expression levels.

### Statistical analysis

The results are reported as the means ± SDs, and the tests were performed a minimum of 3 times. The statistical analysis was performed with SPSS 25.0 software (SPSS, Chicago, IL, USA). A one-way analysis of variance was used to evaluate the statistical significance in three or more groups. However, comparisons of two independent groups were assessed using a two-tailed Student’s t test and chi-square test. The survival rates of each group were analysed via Kaplan‒Meier analysis and log-rank tests, and a *P* < 0.05 was considered significant.

## Electronic supplementary material

Below is the link to the electronic supplementary material.

Supplementary Material 1

## Data Availability

The methods utilized in this study and the availability of data can be obtained from the corresponding author upon reasonable request.
