# Author Correction: lncRNA CHAF1B-2 contributes to the tumorigenesis of gastric cancer by activating the Wnt/β-catenin pathway

**DOI:** 10.1038/s41598-025-91937-6

**Published:** 2025-03-04

**Authors:** Shao-Qi Tian, Jun-Jie Shen, Dao-Ping Sun, Wen-Ming Chen

**Affiliations:** 1 Department of Oncology, Jining Hospital of Xiyuan Hospital of CACMS, Jining, 272011 Shandong Province China; 2 Department of Oncology, Tianchang Traditional Chinese Medicine Hospital, Tianchang, 239300 Anhui Province China; 3 Department of Hematology, Jining No.1 People’s Hospital, Jining, 272011 Shandong Province China; 4 Department of Oncology, Jining No.1 People’s Hospital, Jining, 272011 Shandong Province China

Correction to: *Scientific Reports* 10.1038/s41598-024-84344-w, published online 02 January 2025

The original version of this Article contained an error in Affiliation 1, which was incorrectly given as ‘Department of Oncology, Xiyuan Hospital Jining Hospital, Jining 272011, Shandong Province, China.’ The correct affiliation is listed below:

Department of Oncology, Jining Hospital of Xiyuan Hospital of CACMS, Jining 272011, Shandong Province, China.

The original Article has been corrected.

