# Supplementary material for: lncRNA CHAF1B-2 contributes to the tumorigenesis of gastric cancer by activating the Wnt/β-catenin pathway
Source: Sci Rep. 2025 Jan 2;15:568. doi: 10.1038/s41598-024-84344-w (PMC11695584; doi:10.1038/s41598-024-84344-w)
Supplement: Supplementary file 1 — Supplementary Material 1 [file 41598_2024_84344_MOESM1_ESM.pdf]

lncRNA CHAF1B-2 contributes to the tumorigenesis of gastric cancer via activating the Wnt/β-catenin pathway

AGS

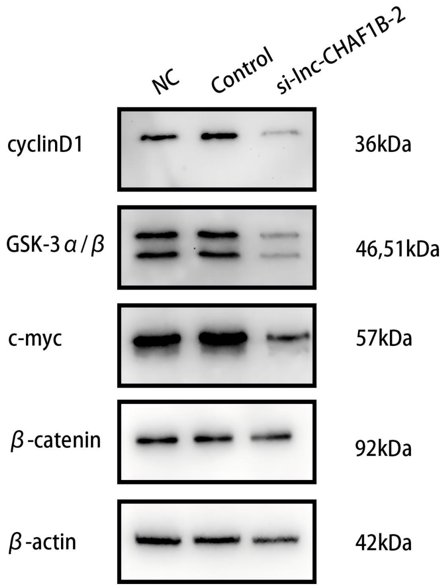
NC
Control
si-lnc-CHAF1B-2
cyclinD1
36kDa
GSK-3α/β
46,51kDa
c-myc
57kDa
β-catenin
92kDa
β-actin
42kDa

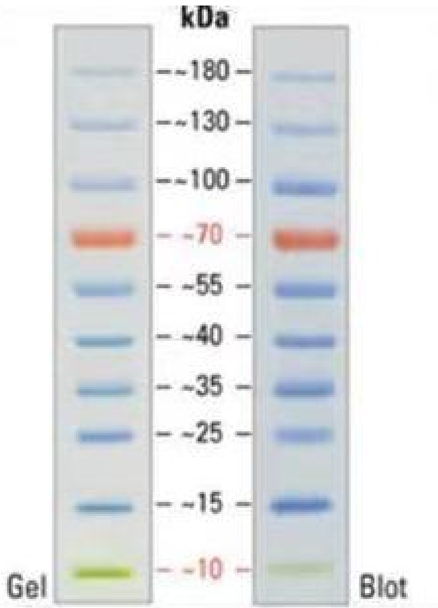
kDa
~180
~130
~100
~70
~55
~40
~35
~25
~15
~10
Gel
Blot

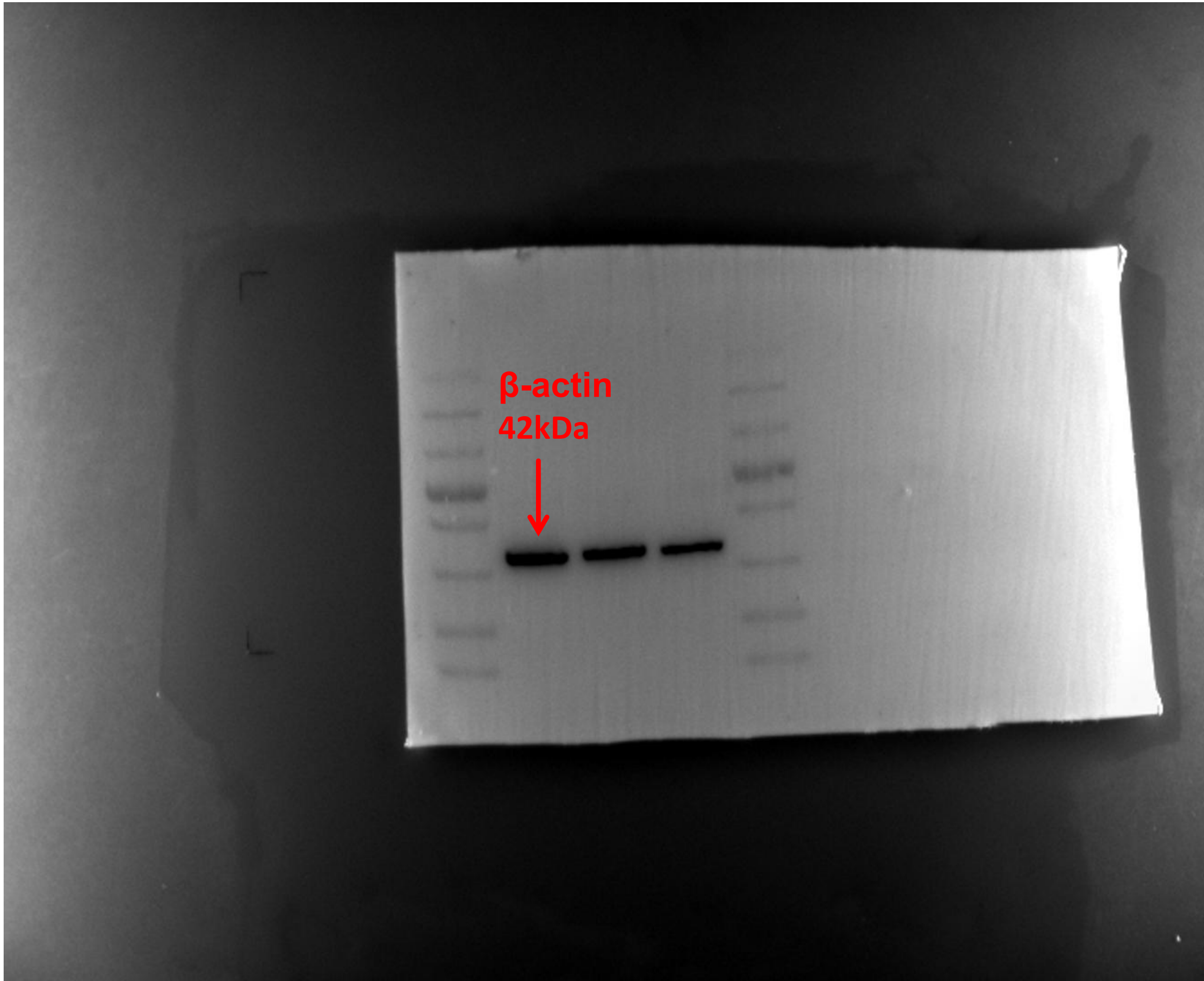
β-actin
42kDa

AGS

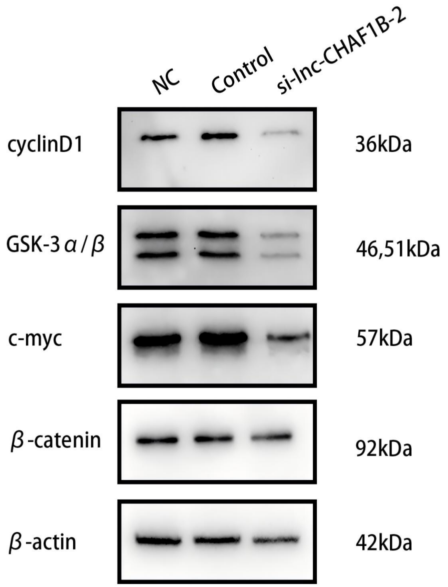
NC
Control
si-lnc-CHAF1B-2
cyclinD1
36kDa
GSK-3 α / β
46,51kDa
c-myc
57kDa
β-catenin
92kDa
β-actin
42kDa

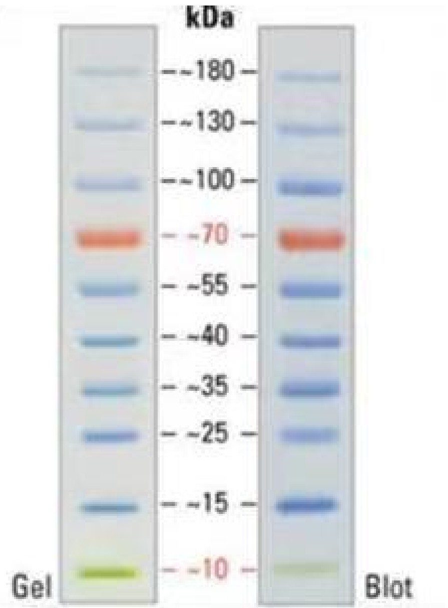
kDa
~180
~130
~100
~70
~55
~40
~35
~25
~15
~10
Gel
Blot

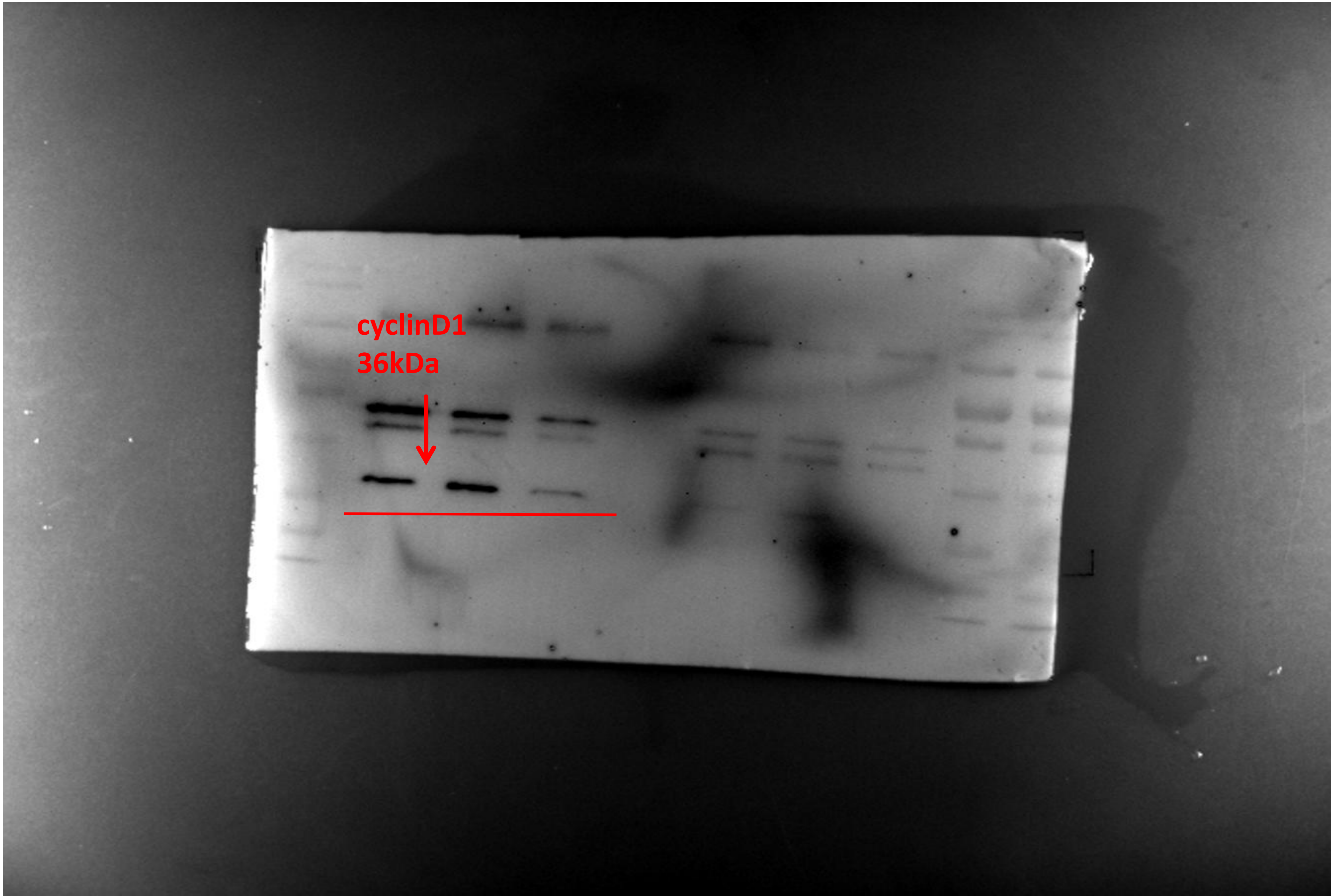
cyclinD1
36kDa

AGS

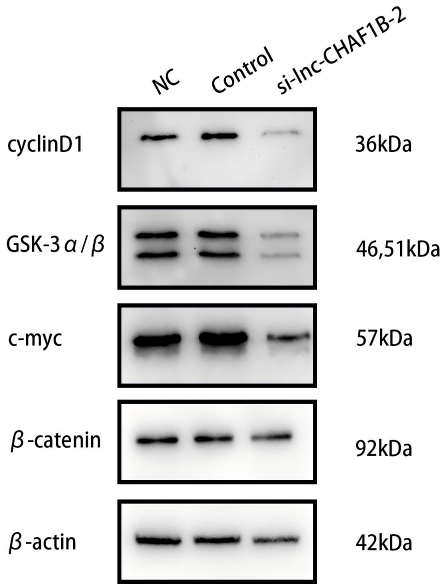
NC
Control
si-lnc-CHAF1B-2
cyclinD1
36kDa
GSK-3α/β
46,51kDa
c-myc
57kDa
β-catenin
92kDa
β-actin
42kDa

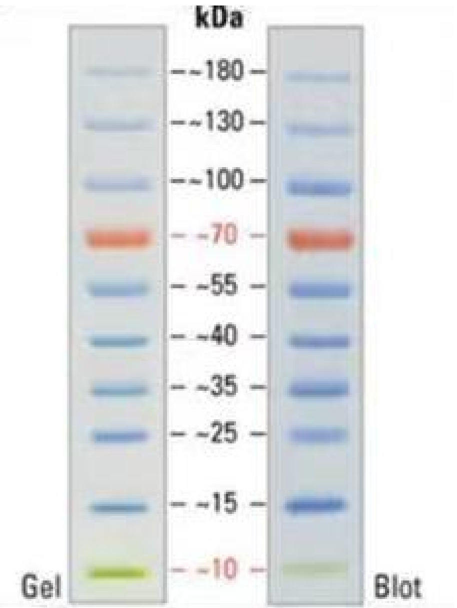
kDa
~180
~130
~100
~70
~55
~40
~35
~25
~15
~10
Gel
Blot

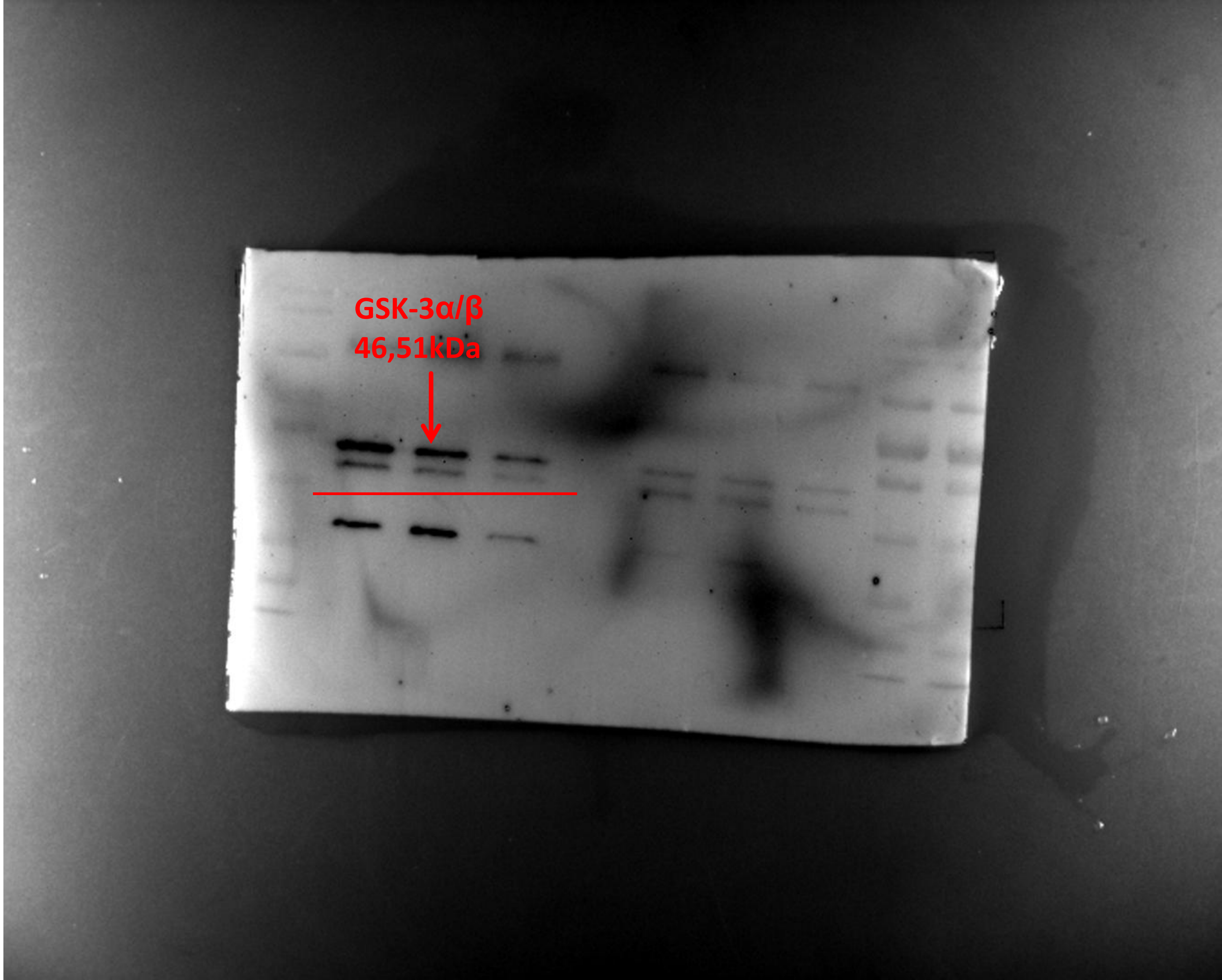
GSK-3α/β
46,51kDa

AGS

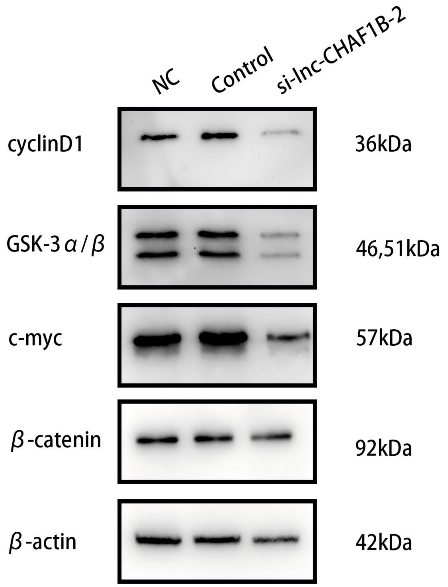
NC
Control
si-lnc-CHAF1B-2
cyclinD1
36kDa
GSK-3α/β
46,51kDa
c-myc
57kDa
β-catenin
92kDa
β-actin
42kDa

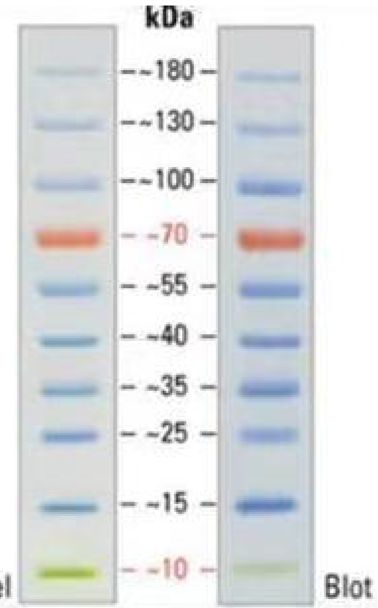
kDa
~180
~130
~100
~70
~55
~40
~35
~25
~15
~10
Gel
Blot

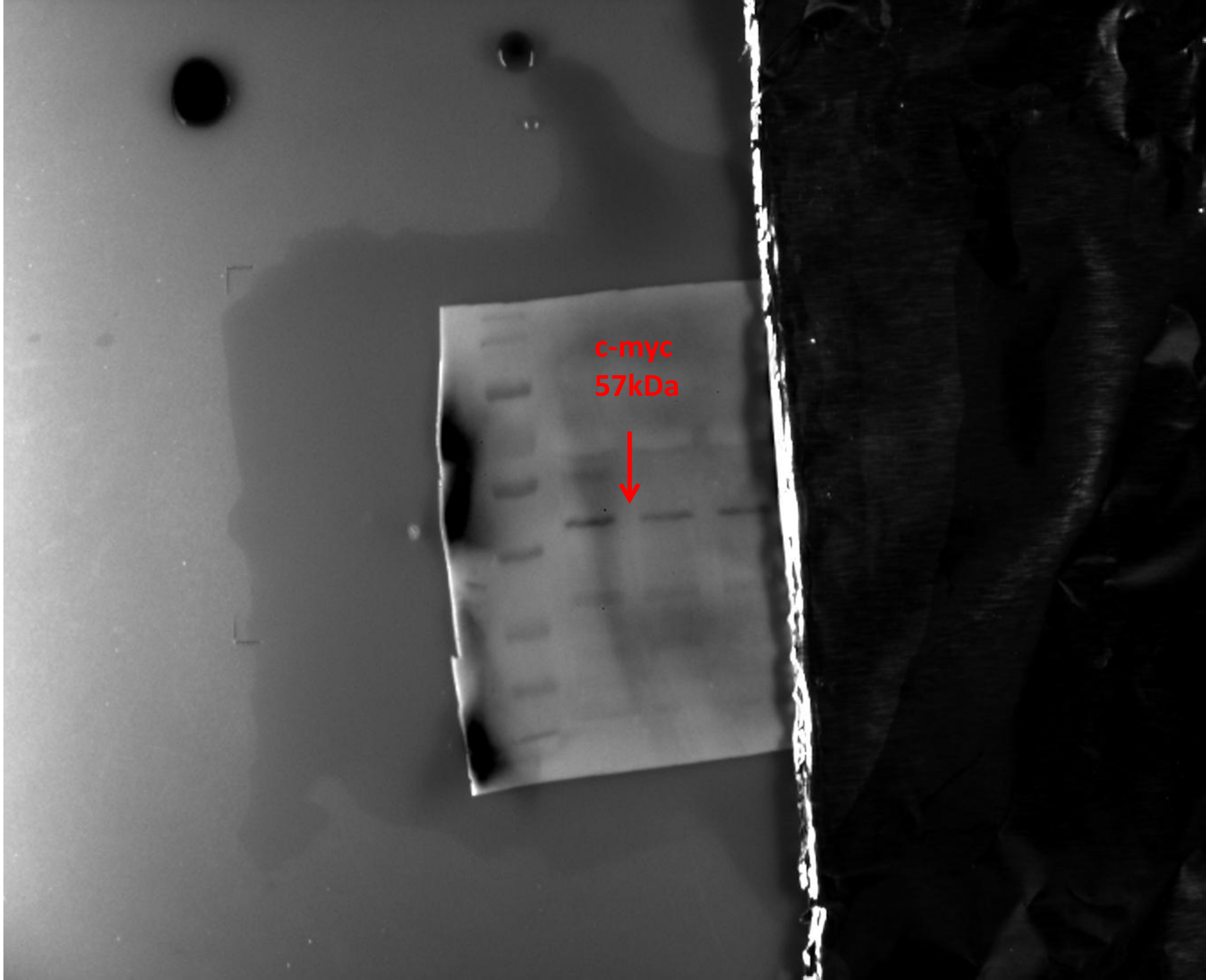
c-myc
57kDa

AGS

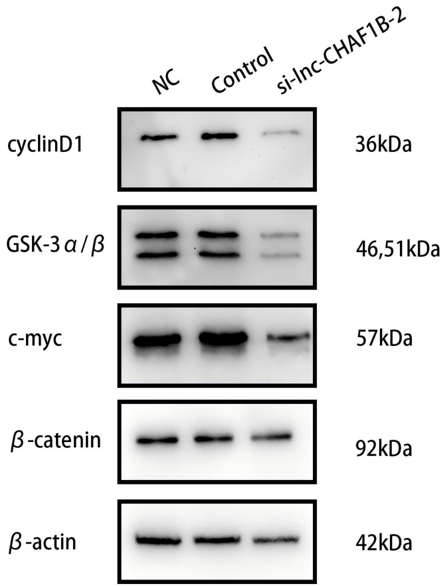
NC
Control
si-lnc-CHAF1B-2
cyclinD1
36kDa
GSK-3α/β
46,51kDa
c-myc
57kDa
β-catenin
92kDa
β-actin
42kDa

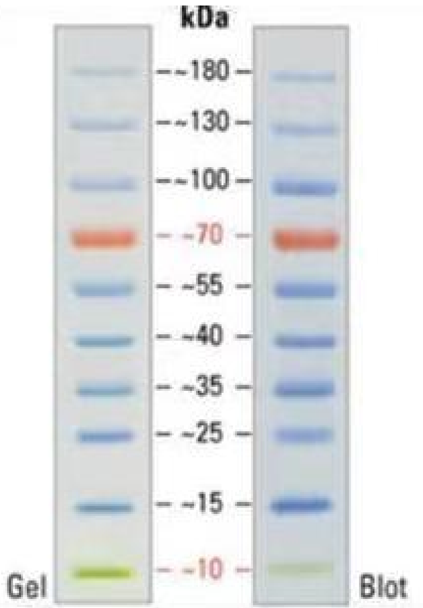
kDa
~180
~130
~100
~70
~55
~40
~35
~25
~15
~10
Gel
Blot

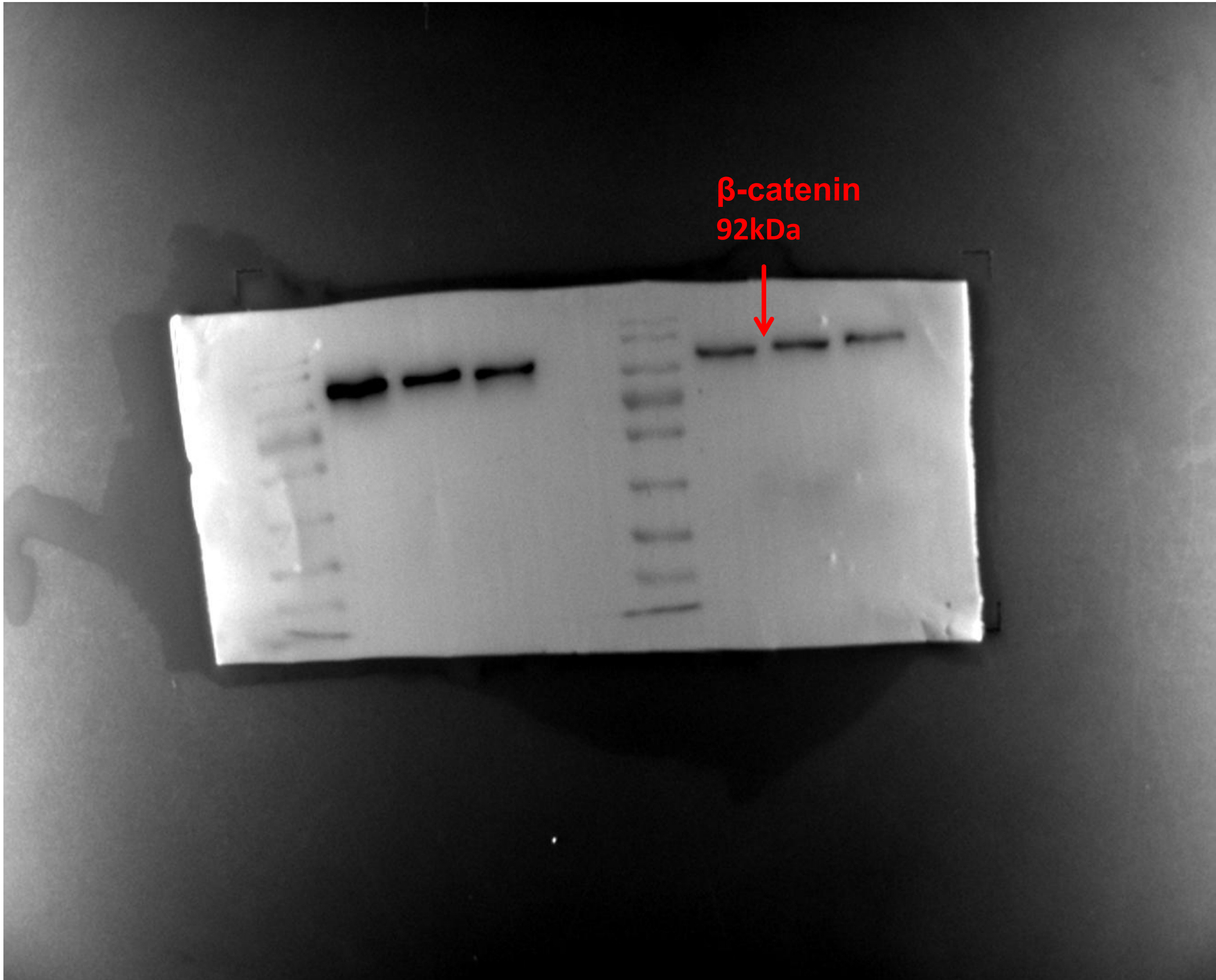
β-catenin
92kDa

HGC-27

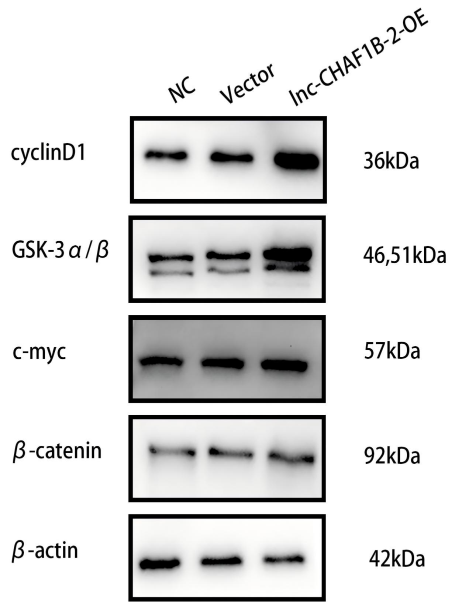
NC
Vector
lnc-CHAF1B-2-OE
cyclinD1
36kDa
GSK-3α/β
46,51kDa
c-myc
57kDa
β-catenin
92kDa
β-actin
42kDa

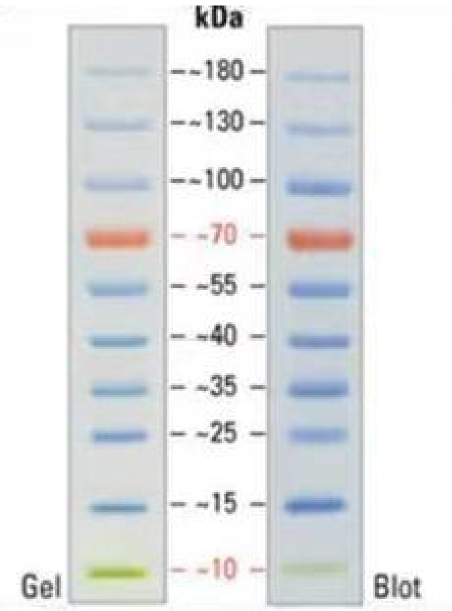
kDa
~180
~130
~100
~70
~55
~40
~35
~25
~15
~10
Gel
Blot

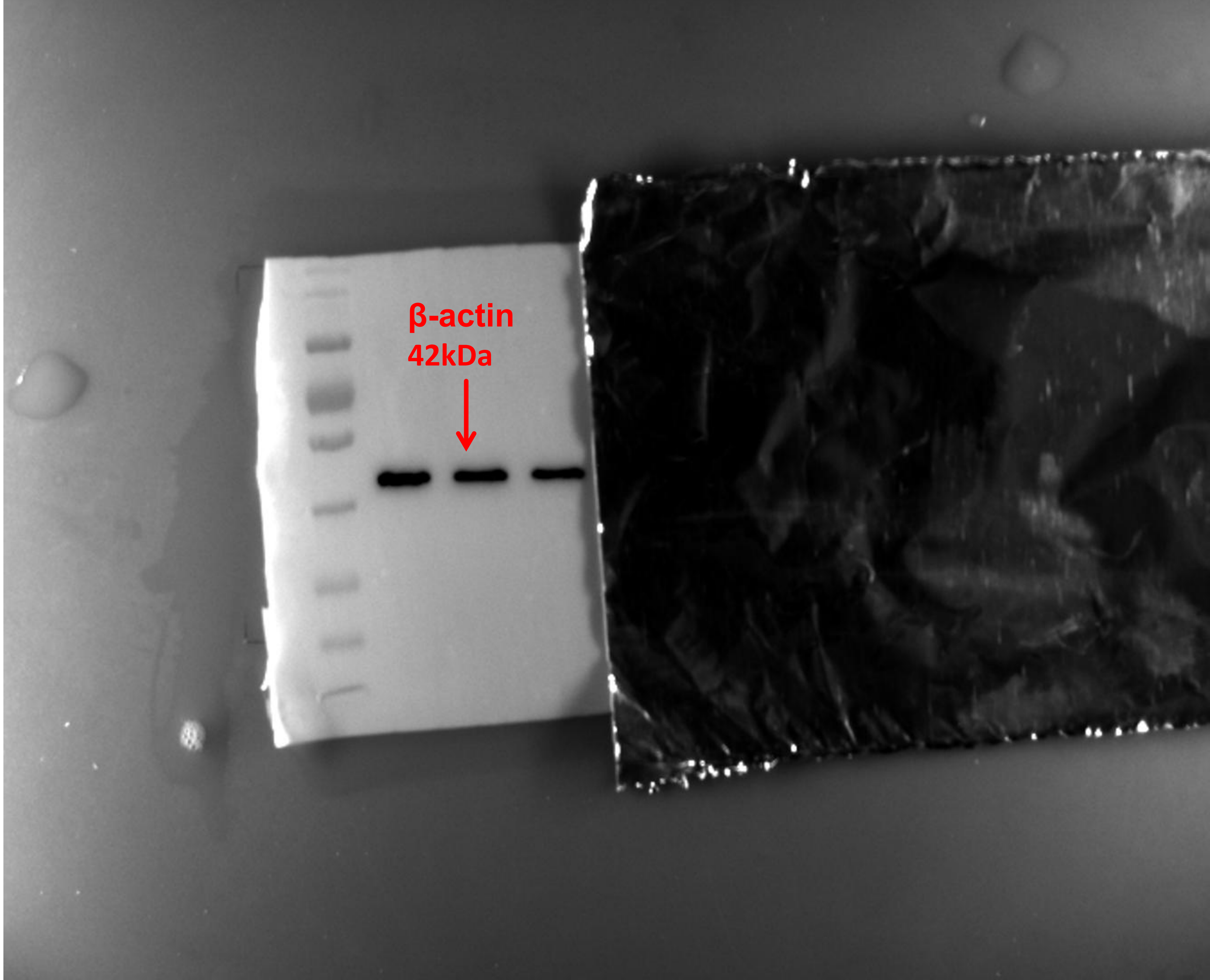
β-actin
42kDa

HGC-27

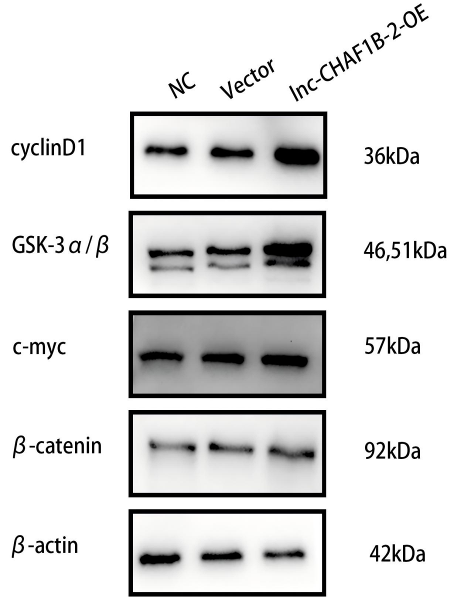
NC
Vector
lnc-CHAF1B-2-OE
cyclinD1
36kDa
GSK-3α/β
46,51kDa
c-myc
57kDa
β-catenin
92kDa
β-actin
42kDa

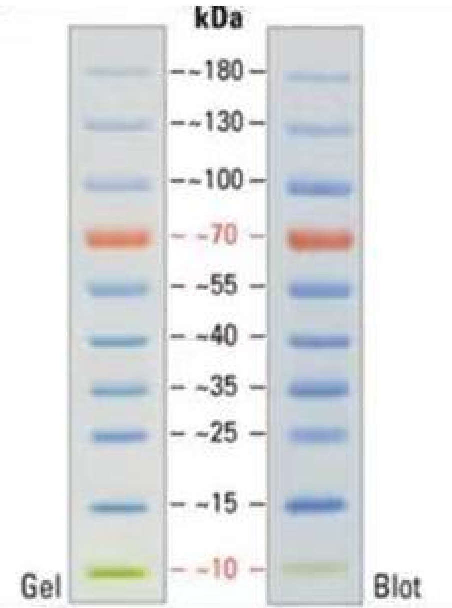
kDa
180
130
100
70
55
40
35
25
15
10
Gel
Blot

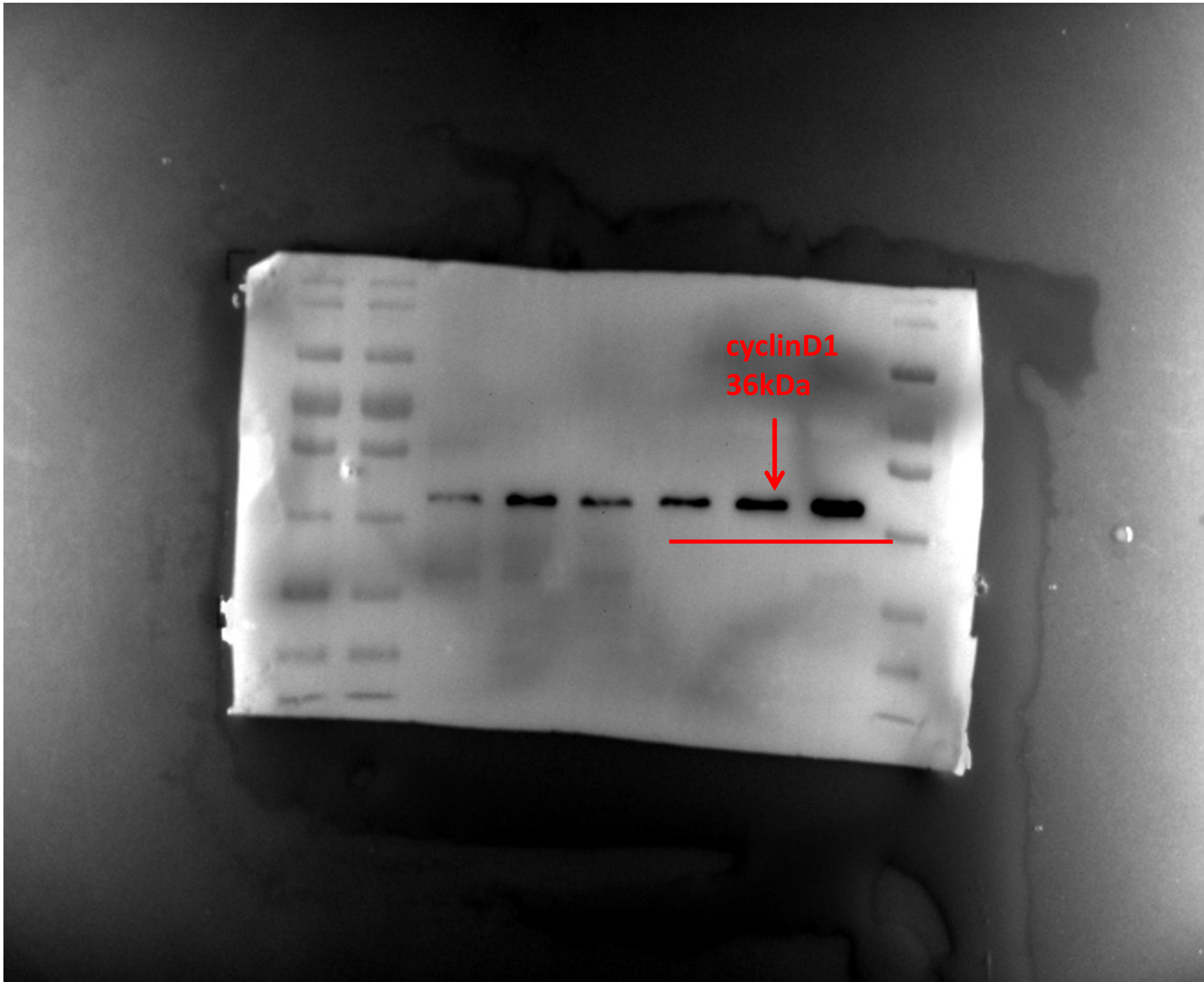
cyclinD1
36kDa

HGC-27

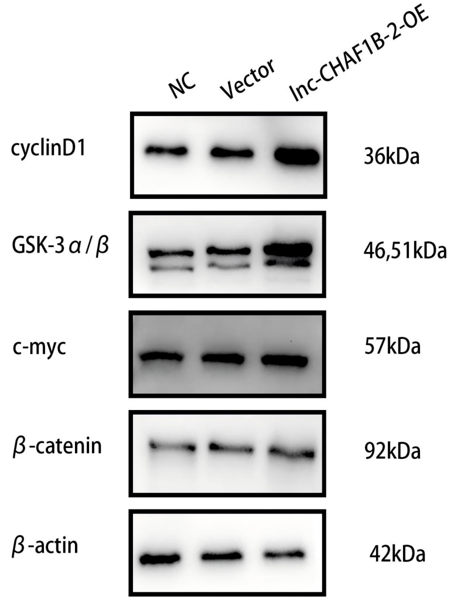
NC
Vector
lnc-CHAF1B-2-OE
cyclinD1
36kDa
GSK-3α/β
46,51kDa
c-myc
57kDa
β-catenin
92kDa
β-actin
42kDa

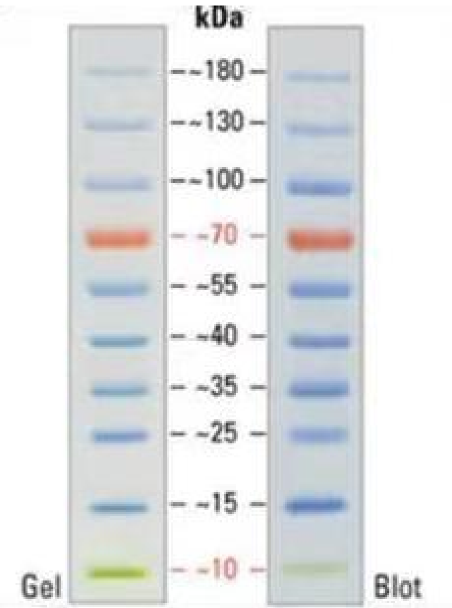
kDa
~180
~130
~100
~70
~55
~40
~35
~25
~15
~10
Gel
Blot

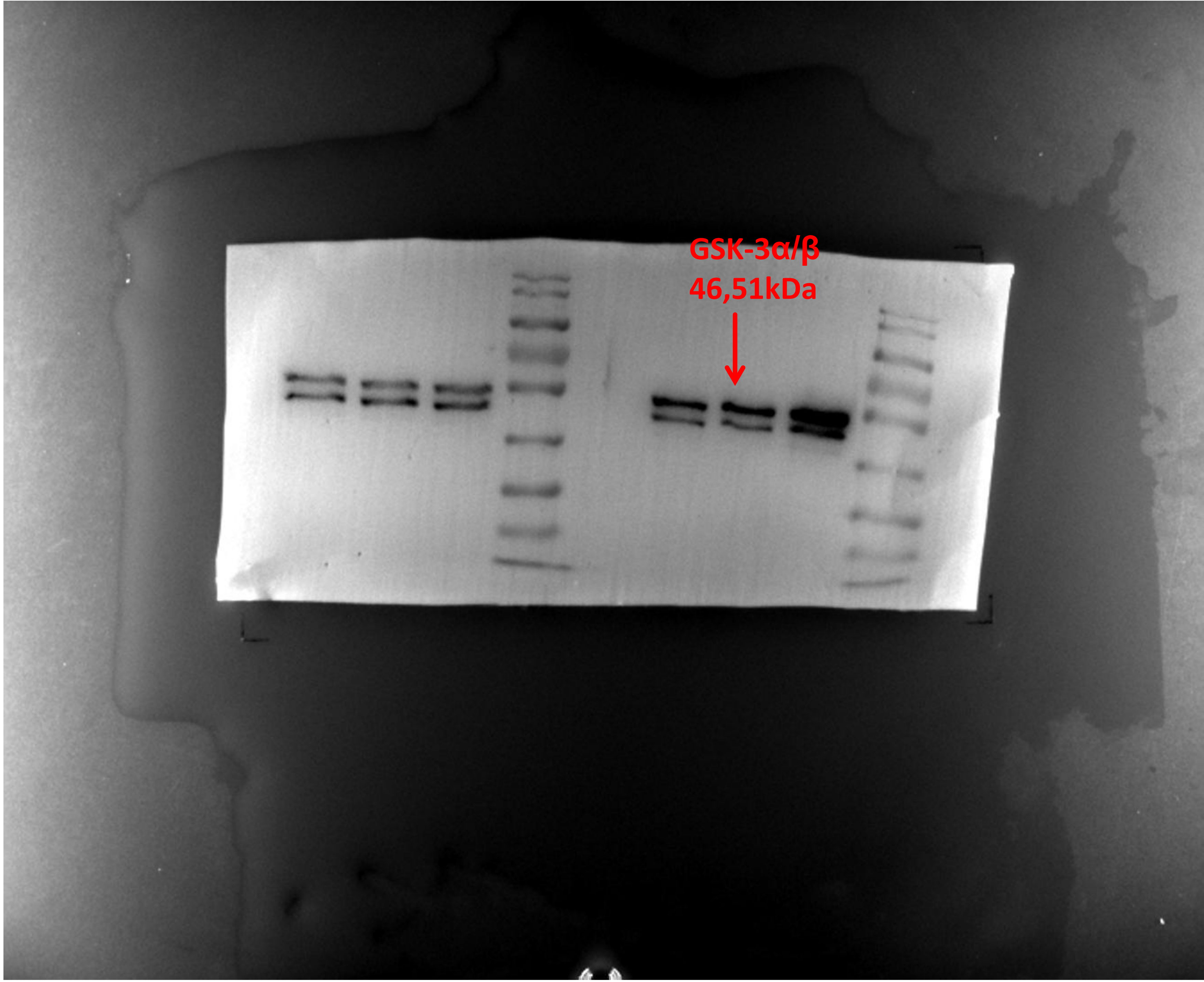
GSK-3α/β
46,51kDa

HGC-27

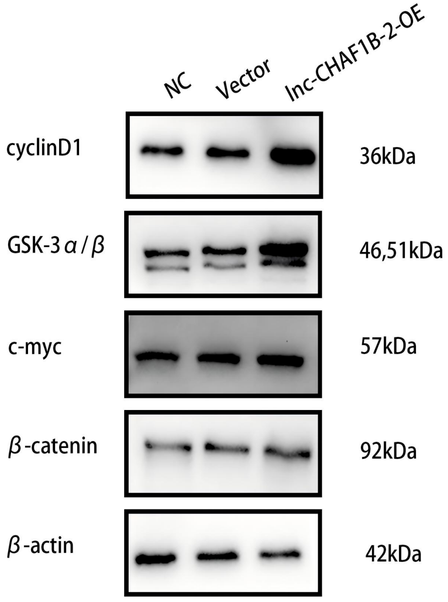

NC
Vector
lnc-CHAF1B-2-OE
cyclinD1
36kDa
GSK-3α/β
46,51kDa
c-myc
57kDa
β-catenin
92kDa
β-actin
42kDa

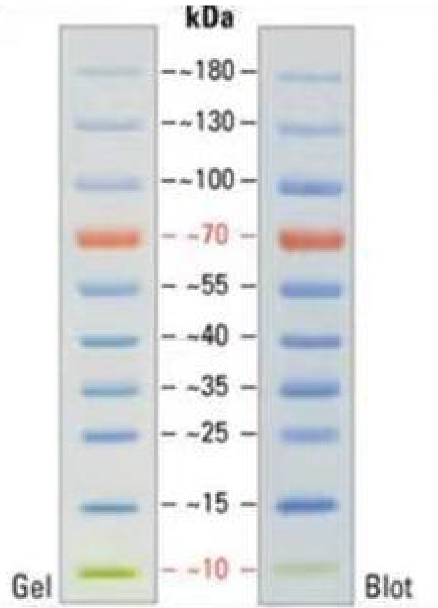

kDa
~180
~130
~100
~70
~55
~40
~35
~25
~15
~10
Gel
Blot

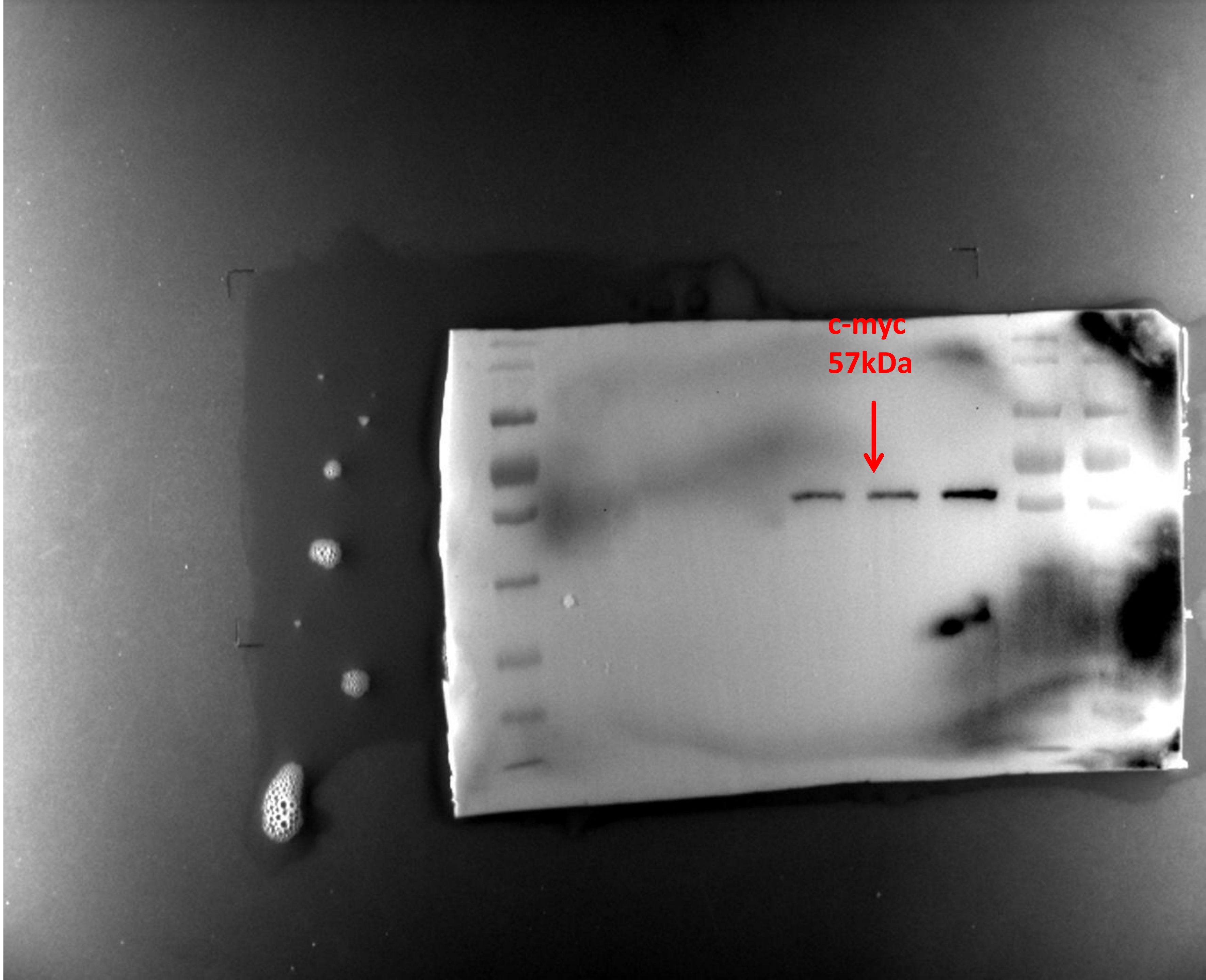

c-myc
57kDa

HGC-27

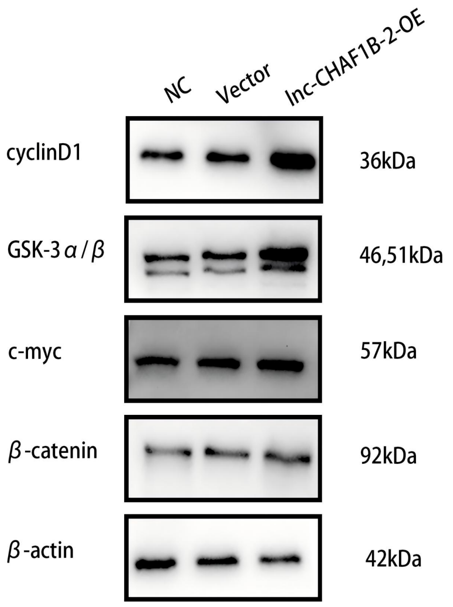
NC
Vector
lnc-CHAF1B-2-OE
cyclinD1
36kDa
GSK-3α/β
46,51kDa
c-myc
57kDa
β-catenin
92kDa
β-actin
42kDa

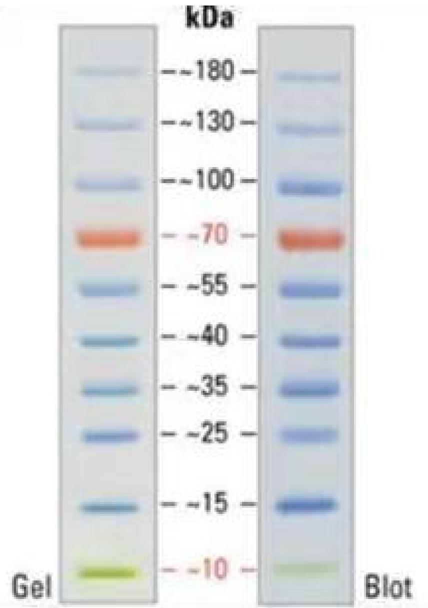
kDa
180
130
100
70
55
40
35
25
15
10
Gel
Blot

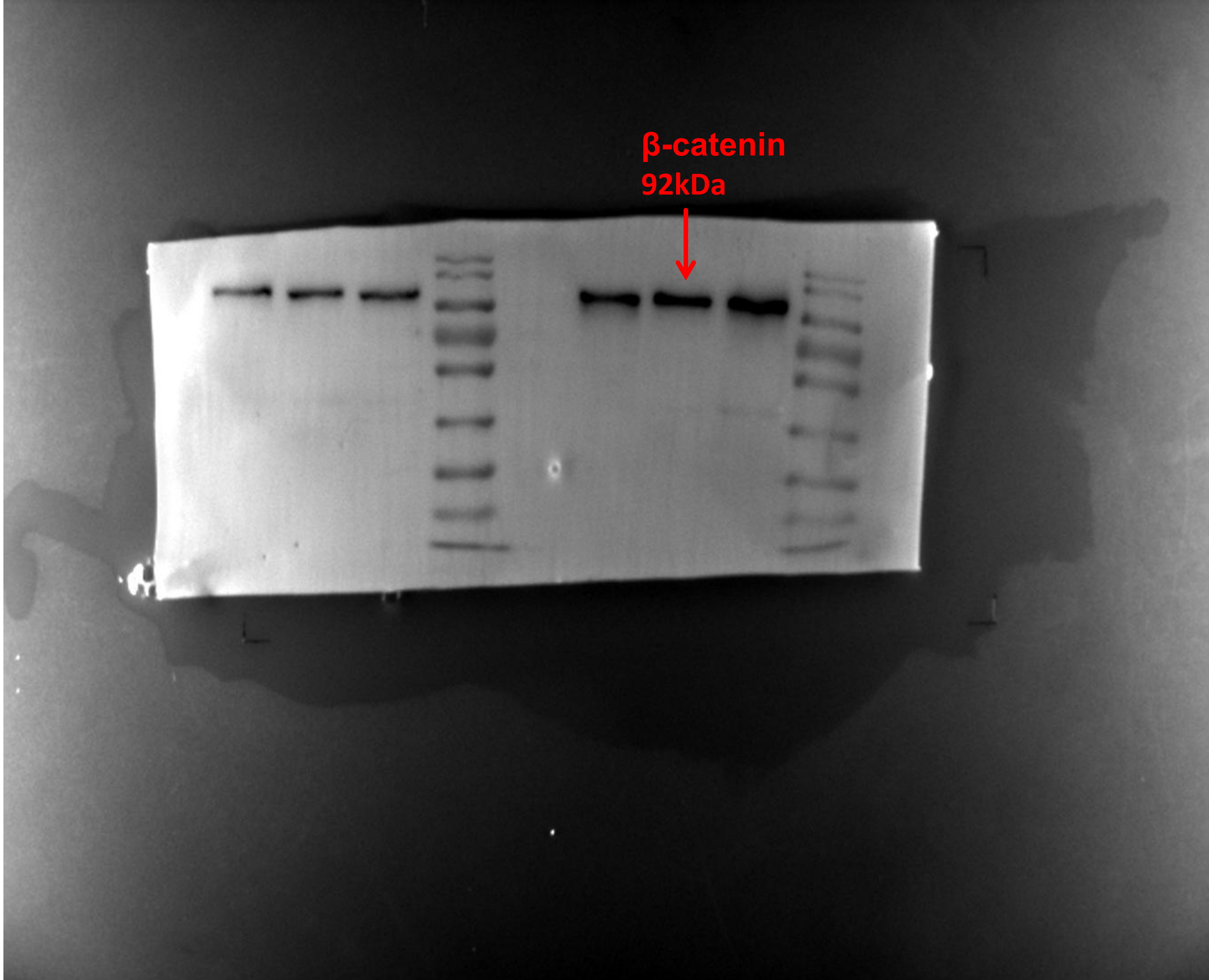
β-catenin
92kDa
